# An Objective Balance Error Scoring System for Sideline Concussion Evaluation Using Duplex Kinect Sensors

**DOI:** 10.3390/s17102398

**Published:** 2017-10-20

**Authors:** Mengqi Zhu, Zhonghua Huang, Chao Ma, Yinlin Li

**Affiliations:** School of Mechatronical Engineering, Beijing Institute of Technology, Beijing 100081, China; vivian_zmq@yahoo.com (M.Z.); huangzh@bit.edu.cn (Z.H.); 20081124@bit.edu.cn (C.M.)

**Keywords:** concussion evaluation, postural stability, balance error scoring system, Kinect sensor

## Abstract

Sports-related concussion is a common sports injury that might induce potential long-term consequences without early diagnosis and intervention in the field. However, there are few options of such sensor systems available. The aim of the study is to propose and validate an automated concussion administration and scoring approach, which is objective, affordable and capable of detecting all balance errors required by the balance error scoring system (BESS) protocol in the field condition. Our approach is first to capture human body skeleton positions using two Microsoft Kinect sensors in the proposed configuration and merge the data by a custom-made algorithm to remove the self-occlusion of limbs. The standing balance errors according to BESS protocol were further measured and accessed automatically by the proposed algorithm. Simultaneously, the BESS test was filmed for scoring by an experienced rater. Two results were compared using Pearson coefficient *r*, obtaining an excellent consistency (*r* = 0.93, *p* < 0.05). In addition, BESS test–retest was performed after seven days and compared using intraclass correlation coefficients (ICC), showing a good test–retest reliability (ICC = 0.81, *p* < 0.01). The proposed approach could be an alternative of objective tools to assess postural stability for sideline sports concussion diagnosis.

## 1. Introduction

Sports-related concussion is common in most sports with a higher incidence in American football, hockey, rugby, soccer, and basketball, of which 78% occur during games as opposed to training [1,2]. The Centers for Disease Control estimates that 1.6 to 3.8 million concussions occur in the US per year in competitive sports and recreational activities [3]. Failure of early recognition and removal of the concussed athlete from play may put the individual at risk for potential complications and long-term consequences [4]. This often requires a rapid and accurate sideline assessment in the midst of competition by certified athletic trainers and team physicians [5].

Since 1997, a multidimensional approach consisting of the systematic assessment of cognition, balance and symptoms has been recommended for the diagnosis and management of sports concussion (SC) [2,6,7]. The multidimensional approach emphasizes multiple diagnostic elements including a physical examination, a survey of post-concussion symptoms, performance-based measures of acute mental status and postural stability, and careful consideration of clinical history [5,8]. Unfortunately, this approach is neither time- nor cost-effective, making them difficult to employ at varying levels of sport. A recent survey of certified athletic trainers indicated that only 21% of respondents used the recommended multidimensional approach to assess SC [9]. When used in isolation, each of the aforementioned clinical measures of cognition, balance, and/or symptoms has been demonstrated to have suboptimal reliability and validity [10,11,12].

As an alternative to the multidimensional approach, the Balance Error Scoring System (BESS) provides an objective measure of balance with the nature of being time- and cost-effective [13]. The BESS relies on the observational skills of trained sports administrator to count the total number of predefined balance errors that a subject makes during three standing stances on firm and foam surfaces. The BESS has been adopted as the current clinical standard of care for balance assessment in concussed athletes on the sideline [14]. However, several studies have addressed the measurement properties of the BESS showing variable inter-rater and test–retest reliability [4,15,16], which are partially based on the raters’ subjective interpretations of errors committed throughout the test and different strictness of the scoring criteria.

To overcome the subjective nature of the BESS, technologies have been used to automate the assessment of postural stability. Such efforts can be divided into two main approaches: postural sway and error scoring [14,17]. The first has been evaluated by using force plate [17,18,19] or wearable devices [14,20,21]. Different from the scores of BESS, the metrics of postural sway are quantified by measured changes in body sway amplitude, velocity, frequency and direction of anterior-posterior, medial-lateral or trunk-rotation movements [20]. The primary clinical balance test for concussion assessment and most often used clinically and described in the literature is the BESS [14]. Therefore, the automated approach of error scoring with increased objectivity and subsequent reliability and validity could have great utility. Brown et al. provided insight into the relationship between inertial sensor-based kinematic outcomes and BESS errors’ scores [22]. Potentially being able to track the six balance errors [23] as they are defined in the BESS standard, the Microsoft Kinect® sensor V1 [24] and V2 [23] have been investigated for the purpose. The use of a single Kinect sensor in the previous studies, however, has been called into the problem of self-occlusion happening when some parts of a human body are hidden. In addition, none of the previous studies have accounted for the error of eye-opening. As such, a large level of variability of the counted errors can result, leading to inaccurate BESS scores. Self-occlusion may be addressed by using multiple Kinect sensors instead of one [25,26,27]. The configuration of two Kinect sensors has been demonstrated to enhance the recognition rate, therefore limiting the issue of self-occlusion [28,29]. Nevertheless, the use of multiple Kinect sensors has not been explored or validated specifically as a way to count the BESS errors.

The aim of the current study is to present and validate a portable, untethered, and affordable solution for sideline sports concussion BESS test using two Kinect sensors. A custom algorithm is developed to automatically score errors committed during each BESS trial from duplex-views. The system is verified by concurrent validity and test–retest reliability in healthy participants. We hypothesized that the use of two Kinect sensors will effectively address the issue of self-occlusion, leading to strong concurrent validity and test–retest reliability when compared to a human rater.

## 2. Methods

### 2.1. Balance Error Scoring System (BESS) and Test Protocol

**Figure 1:**
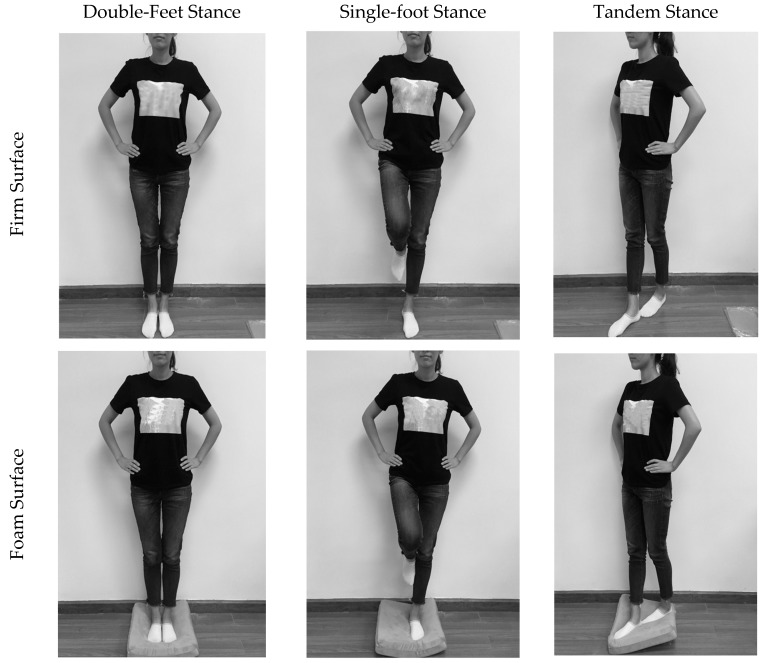
Trials of BESS test in three different stances (double feet, single foot and tandem) on two different surfaces (firm and foam).

The BESS is a clinical accepted measure of postural stability prior to and following a sport concussion. To complete the BESS test, participants are required to maintain balance in three different stances, as shown in Figure 1. Each stance is performed on firm ground and pad form, respectively. The foam pad is medium density and measured 40 cm × 40 cm × 8 cm in size [30]. All trials are 20 s in length. During the completion of each trial, participants are asked to maintain a double leg, single leg or tandem stance with their hands on their iliac crests and with their eyes closed. The BESS errors consist of removal of hands from hips (balance error a), opening of the eyes (balance error b), stepping, stumbling, or falling (balance error c), abduction or flexion of the hip beyond 30° (balance error d), lifting the forefoot or heel off of the firm or foam surface (balance error e), and/or remaining out of the testing position or more than 5 s (balance error f). A maximum of 10 errors could be committed during each trial. If a subject committed multiple errors simultaneously, only one error was recorded. For example, if a subject stumbled, removed his or her hands from their hips and opened their eyes simultaneously, only one error is counted [13]. The balance error is identified by human skeleton data from two Kinect sensors and the final test score is counted. Testing consists of two parts separated by 7 days.

To complete the BESS, subjects are asked to stand at 2.5 m away from the sensors. Each participant is then instructed on how to complete the BESS. Following instruction and assurance of participant understanding, each participant completes the BESS test as previously described. After each trial, a 30 s rest period is employed. An experienced rater (over 60 h of grading experience) simultaneously counts the number of BESS errors for each trial. All trials are video recorded for a follow-up proof counting in order to ensure the accuracy of error numbers. The same testing protocol is administered seven days after the first session.

### 2.2. Instrumentation and Configurations

**Figure 2:**
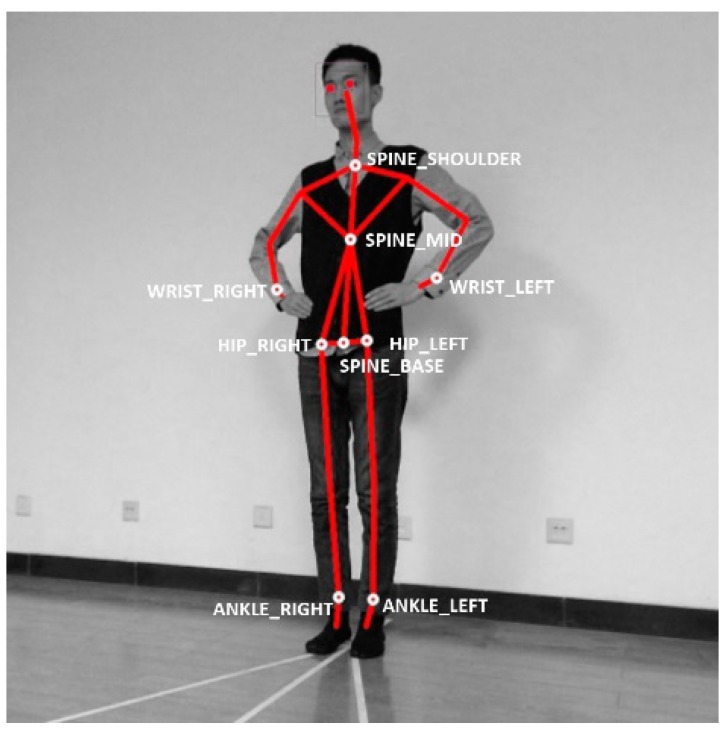
The selected joints used for the BESS test.

The Kinect V2 sensors (Microsoft Corporation, Redmond, WA, USA) were deployed to measure the 3D coordinates of the skeletons and joints of the human body, which were then being used to judge the aforementioned balance errors a–f using custom-made algorithms. Though the camera is capable of obtaining 25 human skeletal joints through the depth image, only wrists, hips, ankles, spine, shoulder and hip center are used in the method, as denoted by the nine white circles in Figure 2. Experimental equipment includes two Kinect V2 cameras and two laptop computers with Windows 8.1 operating system (Microsoft Corporation, Redmond, WA, USA), Intel Core i5 processor and 4 GB memory. The algorithms were implemented in Visual Studio 2013 (Microsoft Corporation, Redmond, WA, USA) and Kinect SDK 2.0 (Microsoft Corporation, Redmond, WA, USA).

The basic configuration consideration for a Kinect sensor is to put the subject conducting the trial stances in the field of view (FOV), even when the movement of the hip is up to 30° of abduction. Moreover, the nine skeletal joints, as denoted in Figure 2, should not be occluded by body parts in the camera vision, or there should be no self-occlusion, for all three of the stances.

**Figure 3:**
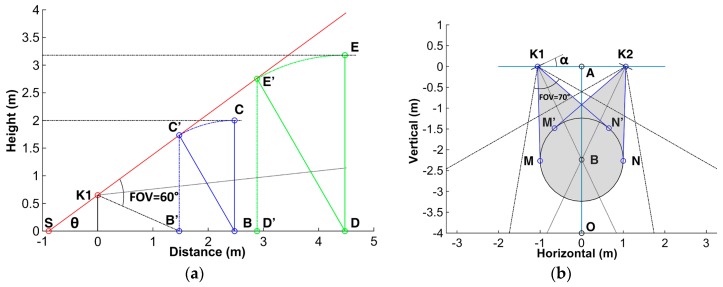
Illustration of duplex Kinect sensor placements. (**a**) the vertical field of view (FOV) of single Kinect sensor and setup for a 2 m high subject; (**b**) the positions, horizontal FOVs and required space (gray color) of the two sensors.

The vertical FOV of a single Kinect is up to 60°, illustrated as angle ∠C’K1B’ in Figure 3a; Point K1 represents the Kinect sensor mounted at height of 65 cm, which is regarded as the ideal operating value of Microsoft Xbox One floor mounting stand; Point B is the test position where the subject stands, and line BC denotes the height of subject, herein taking 2 m as the representative value.

When the subject leans the hip maximally to 30°, the projection height in the vertical axis is under the curve CC’ in math. The poin*t* C’ corresponds to the subject’s head position at the abduction angle 30°, which determines the upward tilt angle of the Kinect sensor and the minimal camera to subject distance (MCSD) that is able to watch the whole body skeletons. Under the prescribed condition, the distance is computed to be 2.48 m and the radius BB’ of the test area is 1 m mathematically. The duplex sensor setup in horizontal view is shown in Figure 3b, in which *K1* and *K2* denote the two Kinect sensors. The distance between the two sensors is 2.12 m and the required test space as illustrated by a gray color in Figure 3b is 5.21 square meters. Since the horizontal FOV of the camera is 70°, the horizontal rotation angle of the camera, *α*, is allowed to be between 15° and 36°.

Although the above configurations are determined supposing 2 m as the height of participant, the sensor can track the whole body skeletons of shorter subjects as well, without any alteration of the setup parameters. When the subject is taller than 2 m, the mathematical expression between the MCSD value *d* and the subject height h can be described as

(1) $$\{\begin{array}{c} d=\left(\frac{\sqrt{3}}{2}\cot\;\theta +0.5\right)\times h-H_{s}\cot\;\theta \\ \frac{\cot\left(60^{{}^{\circ}}-\theta \right)}{\cot\theta }=\frac{\frac{\sqrt{3}}{2}H_{r}-H_{s}}{H_{s}} \end{array},$$

where θ is the angle ∠C’SB’ as denoted in the Figure 3a, $H_{s}$ and $H_{r}$ are the sensor mounting height of 0.65 m and the reference subject height of 2 m, respectively. Equation (1) is obtained according to the triangular relationships of the sensor setup, as shown in Figure 3a, in which the setup parameters including the tilt angle, horizontal rotation angle and mounting height of the sensors are all the same as the case of the 2 m high subject. Typical numerical solutions of Equation (1) are present in Table 1. In the situation, the subject would simply move a distance referring to Table 1, along line AO toward point O as denoted in Figure 3b. The fixed installation parameters will help to ease the use of the system.

It is noteworthy that the above setup parameters are the worst case values. For example, there is less chance that one can fall like a rigid body without flexing one’s hip, and hence the test area diameter should be less than 2 m. Therefore, in a real situation when these values are smaller, the system will have better performance in terms of required installation space and MCSD distance.

### 2.3. Algorithm Development

Microsoft Kinect SDK 2.0 provides all the methods or functions to acquire the skeletal joints coordinates of recognized users. In the tracking loop, *NuiSkeletonTrackingEnable* method is called to enable the tracking and *NuiSkeletonGetNextFrame* method is to access the members of the *NUI_SKELETON_FRAME* structure to receive information about the users. In the members of the skeleton frame structure, the *NUI_SKELETON_DATA* structure has a *SkeletonPositionTrackingState* array, which contains the tracking state for each joint. A tracking state of a joint can be “tracked” for a clearly visible joint, “inferred” when a joint is not clearly visible and Kinect is inferring its position, or “non-tracked”. In addition, the *SkeletonPositions* array in the skeleton frame structure contains the position of each joint.

Once the positions and tracking states of the participants are obtained, the data from two Kinect sensors will be fed into a custom-made algorithm to compute BESS scores. It is mainly composed of two parts: error motion recognition and scoring algorithm.

#### 2.3.1. Error Motion Recognition Algorithm

The error motion recognition algorithm (EMRA) functions to determine whether the balance errors occur during the BESS test. Specifically, the EMRA obtains the coordinates of skeletal joints from the two Kinect sensors and then extracts the feature vectors of the predefined balance errors, which is further processed by comparing the deviation of the joints away from its original position. Subsequently, the EMRA merges the results of two cameras by weighting a fusion coefficient in order to choose the best candidate of the skeletal joints from the two Kinect sensors, especially when one is self-occluded or incurred poor accuracy. Supposing the coordinate of a joint at time *t* is $p_{i}^{t}=(x_{i}^{t},y_{i}^{t},z_{i}^{t})$, the vector between joint *i* and joint *j* at time *t* can be expressed as $p_{i,j}^{t}=p_{i}^{t}-p_{j}^{t}$. Then, the error equation $\delta^{t}(i,j)$ for balance error a, c, e is described as follows:

(2) $$\delta^{t}(i,j)=\left\{ \begin{array}{lll} 1, & & \left\|p_{i}^{t}-p_{j}^{t}\right\|>H,\left\|p_{i}^{t-1}-p_{j}^{t-1}\right\|\leq H\\ 0, & & otherwise \end{array}\right.,$$

where *H* is the predefined threshold. When the $\delta^{t}(i,j)$ is computed to be 1, a balance error is found. In the case of balance error a, the $p_{i}^{}$ and $p_{j}^{}$ are the coordinates of the joint wrist and hip, respectively; the term $\left\|p_{i}^{t}-p_{j}^{t}\right\|$ is $\left\|p_{wrist}^{t}-p_{hip}^{t}\right\|$, and the *H* equals to $\left\|p_{wrist}^{0}-p_{hip}^{0}\right\|$, which means the distance between joint wrist and hip at the initial time. For the balance error c, the $p_{i}^{}$ is joint spine_mid and $p_{j}^{}$ is its initial value, and the term $\left\|p_{i}^{t}-p_{j}^{t}\right\|$ is then $\left\|p_{spine\_mid}^{t}-p_{spine\_mid}^{0}\right\|$. For the balance error e, the $p_{i}^{}$ is joint ankle and the term $\left\|p_{i}^{t}-p_{j}^{t}\right\|$ becomes $\left\|p_{ankle}^{t}-p_{ankle}^{0}\right\|$. The threshold *H* is set to 10 cm in the study for balance error c and e, the amount of which is determined by experiments and set to be large enough to reflect slight balance error motion meanwhile suppressing the jitter caused by the camera vision noise.

The error equation of balance error d is defined by:

(3) $$\delta^{t}(i,j)=\left\{ \begin{array}{lll} 1, & & \cos(p_{i,j}^{t-1},v)>H,\cos(p_{i,j}^{t},v)\leq H\\ 0, & & otherwise \end{array}\right.,$$

where $p_{i,j}^{}$ is the vector from joint spine_base to spine_shoulder, *v* means a vertical vector, and threshold *H* is 0.866 corresponding to the cosine 30° of the maximal allowed hip flexion angle.

The error recognition fusion equation is expressed as:

(4) $$\varphi^{t}=\left\{ \begin{array}{lll} 1, & & \omega_{A}^{t}(i,j)\cdot \delta_{A}^{t}(i,j)+\omega_{B}^{t}(i,j)\cdot \delta_{B}^{t}(i,j)\geq 1\\ 0, & & \omega_{A}^{t}(i,j)\cdot \delta_{A}^{t}(i,j)+\omega_{B}^{t}(i,j)\cdot \delta_{B}^{t}(i,j)<1 \end{array}\right.,$$

where $\delta_{A}^{t}(i,j)$ and $\delta_{B}^{t}(i,j)$ represent the results of error recognition at time *t* from Kinect sensor *A* and *B*, and $\omega_{A}^{t}(i,j)$ and $\omega_{B}^{t}(i,j)$ are the weight coefficients of sensor *A* and *B* at time *t*, respectively. The values of $\omega_{A}^{t}(i,j)$ and $\omega_{B}^{t}(i,j)$ are determined according to the capture state of joint (well tracked, inferred and not tracked), which are provided by the Microsoft Kinect SDK. The values of ω(i,j) are shown in Table 2.

As for the balance error b, it can be achieved directly by calling the *GetFaceProperties* function of the Microsoft Kinect SDK to recognize the states of the eye (open, closed and unknown).

#### 2.3.2. Scoring Algorithm

In addition to the error motion recognition, the software should also be able to score the BESS trials automatically. As one error might be accompanied by multiple simultaneous or subsequent errors, redundant errors count should be screened out. For the balance errors *α* and *β*, $t_{i,begin}^{\alpha }$ is the start time of the *i*-th occurrence of the balance error *α*, and the end time is $t_{i,end}^{\alpha }$; $t_{j,begin}^{\beta }$ is the start time of the *j*-th occurrence of the balance error *β*, and the end time is $t_{j,end}^{\beta }$. Whether the balance error *α* and *β* occur simultaneously is determined by the following equation:

(5) $$\left\{ \begin{array}{l} t_{i,begin}^{\alpha }-t_{j,begin}^{\beta }<0\\ t_{i,end}^{\alpha }-t_{j,begin}^{\beta }\geq 0 \end{array}\right.\;\;\;or\;\;\;\left\{ \begin{array}{l} t_{j,begin}^{\beta }-t_{i,begin}^{\alpha }<0\\ t_{j,end}^{\beta }-t_{i,begin}^{\alpha }\geq 0 \end{array}\right..$$

If simultaneous errors are detected, the later one will be ignored.

A total of six stances are performed in sequence (double feet, one foot, tandem) on the firm surface followed by the foam surface. For each stance, there are six types of balance errors a–f. The equation of BESS test score at *j*-th stance is defined as follows:

(6) $$Score_{j}=\left\{ \begin{array}{lll} \sum_{i=1}^{6}\varphi_{i}-\gamma , & & 0\leq \sum_{i=1}^{6}\varphi_{i}-\gamma +\varepsilon <10\\ 10, & & \sum_{i=1}^{6}\varphi_{i}-\gamma +\varepsilon \geq 10 \end{array}\right.,$$

where $\varphi_{i}$ is the number of occurrences of balance error i, and γ represents the number of simultaneous errors that should be ignored. The constant ε is to indicate whether or not the subject fails to maintain the testing stance less than 5 s or remains out of a proper testing position for longer than 5 s. If so, ε is 0; otherwise, ε is 10. The maximum score of each stance is limited to 10, and the total BESS score is the sum of $Score_{j}$ counted during all six stances. The total score acquired automatically using the above algorithm is then compared with the rater score to verify the Duplex Kinects System’s validity and reliability.

### 2.4. Subjects

The current study was approved by the institutional academic board. Thirty healthy and physically active subjects (12 female and 18 male) between the 22 and 31 years (yr) of age (25.6 ± 2.56 yr), and who were 158 to 190 cm tall (171.1 ± 6.72 cm) participated in the current study. Exclusion criteria included neurological or musculoskeletal conditions, respiratory or cardiovascular problems, and pregnancy. All subjects were informed of the purpose, methods and instructions to complete the BESS and signed informed consent.

### 2.5. Analysis

Concurrent validity of scores obtained by the custom-made duplex Kinect BESS software and by the rater were assessed using Pearson correlation coefficients. In addition, intraclass correlation coefficients ICC (2,1) (2-way random effect, single measure model) were used to assess the test–retest reliability of the custom-made duplex Kinect BESS software between days 1 and 8. A modified version of BESS (mBESS) using only three stance conditions on the firm surface, which is currently included in the SCAT3 protocol and the Official NFL Sideline Tool [31], has also been accessed. All analyses were conducted with *p* < 0.05 as the significance level and performed using SPSS Version 20.0 (IBM Corporation, Armonk, NY, USA). For the Pearson coefficient *r*, it was excellent relationship if *r* was greater than 0.90, good relationship if *r* was between 0.8 and 0.89, a fair degree if *r* was between 0.7 and 0.79, and poor if r was below 0.70 [32]. Regarding the ICC coefficients, it was excellent if ICC was greater than 0.90, good if ICC was between 0.75 and 0.90, moderate if ICC was between 0.50 and 0.75, and poor if ICC was below 0.50 [33]. 

## 3. Results

**Figure 4:**
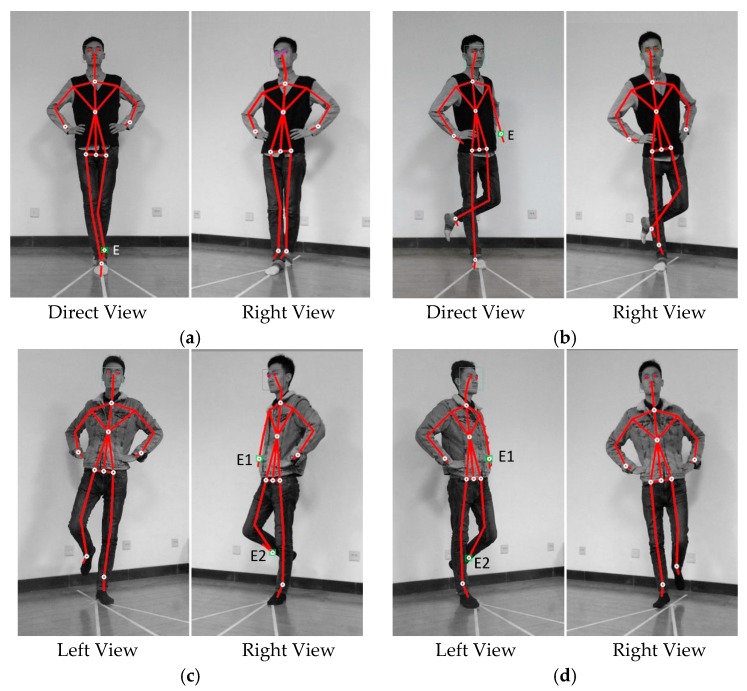
Representative self-occlusion images in direct, left or right view of Kinect sensors. (**a**) in the direct view, the rear ankle joint E is occluded whereas not in the right view; (**b**) in the direct view, the left wrist joint E is occluded when the body twisted, whereas not in the right view; (**c**) in the right view, the joints E1 and E2 are occluded, whereas not in the left view; (**d**) in the left view, joints E1 and E2 are occluded whereas not in the right view.

In order to validate the self-occlusion of the proposed sensor configuration, representative images captured by sensors are shown in Figure 4. Direct view indicates the image captured by the sensor placed at position A in Figure 3b, facing directly to the subject; left and right view shows images captured by sensors placed at position K1 and K2 in Figure 3b, respectively, facing to the subject in the same way as depicted in Figure 3b. In Figure 4a, the rear ankle is occluded by the front one during tandem stance in direct view, resulting in a self-occlusion joint denoted as E in the figure. However, the rear ankle joint occluded in direct view is tracked properly in the right view. The same result could be found in the other three stance situations. The red dots overlaid on the eyes were generated by software automatically in case the eyes were tracked by the sensor, as shown in Figure 4.

Further experiments were performed to examine the performance of the system for the purpose gof the BESS test. Table 3 shows the statistical results of the system measurements and the rater counting of the six balance conditions. In each condition, balance errors a–f committed by the subject were counted, including the error b of eye opening. Concurrent validity of the system and the rater counting shows that the system’s BESS total score is 11.83 ± 7.62, and the rater’s is 11.33 ± 7.89, and the Pearson coefficient *r* is 0.93 (*p* < 0.05). The total mBESS scores counted by system and rater are 4.67 ± 3.13 and 4.43 ± 3.40, respectively, indicating that the system score accurately fits the rater’s (*r* = 0.92, *p* < 0.05) in the subset of balance conditions (firm surface only).

**Figure 5:**
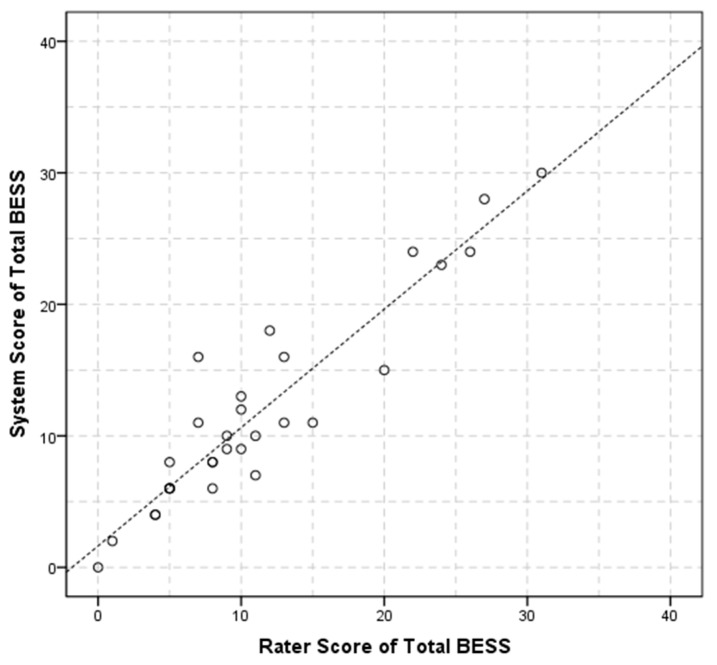
BESS score for rater and system.

A scatter plot is also presented in Figure 5, indicating that the system and rater score correlated positively and agreed with each other.

**Figure 6:**
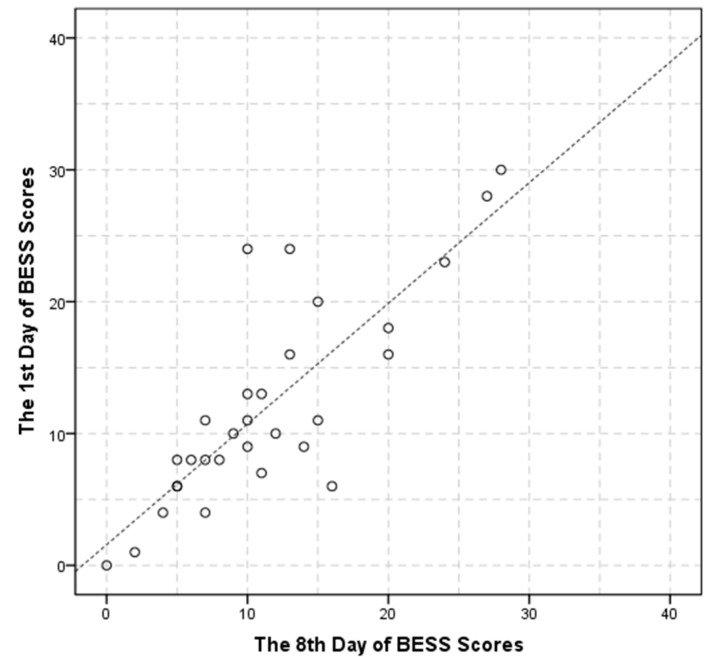
BESS score for day 1 and day 8.

Test–retest reliability of the system measurements on the first and eighth day as well as the ICC value in each condition shows that the first day total score of the BESS test is 12.07 ± 7.75, the eighth day total score of the BESS test is 11.47 ± 6.93, and the ICC was 0.81 (*p* < 0.001). The mBESS test score is 4.73 ± 3.17 on day 1 while 4.70 ± 3.76 on day 8, with ICC value of 0.84 (*p* < 0.001). The detail is illustrated in Table 4. The scatter plot of the BESS score for the first day and the eighth day is shown in Figure 6.

## 4. Discussion

The BESS is recognized as the current standard for the evaluation of sports related concussion. However, the intra- and inter-raters reliability of BESS scores has been questioned. In an attempt to overcome the subjective limitations of the BESS, the proposed method used two Kinect sensors along with a custom-made algorithm to track the postural balance errors committed by the participant. The primary findings derived from the results include: (1) the duplex views from two Kinect sensors can compensate for each other and track the key human body skeletal joints without blind spot or self-occlusion, even during the challenging tandem stance; and, (2) due to the constraint of the sensor’s field of view, placement parameters including sensor separation distance, tilt angle and mounting height, etc., should be properly determined by taking into consideration the portability, installation space and ease of setup.

Our proposed custom-made algorithm for duplex Kinect BESS yielded excellent correlation coefficients to the human rater’s BESS scores (*r* = 0.93, *p* < 0.05) and test–retest reliability (ICC = 0.81, *p* < 0.05) across an eight-day test–retest interval. Our method has greater test–retest reliability compared to that of human rater’s BESS scores present by Finnoff et al. (ICC = 0.74) [16] and by Valovich et al. for high school participants (ICC = 0.70) [34]. These results indicate that the suggested duplex Kinect BESS method may be an objective and reliable measure of postural stability.

Brown et al. employed inertial sensors to evaluate the oBESS scores using a custom-made equation in an effort to overcome the subjectivity of the BESS scoring system [22]. Even though their oBESS was able to produce scores with accurate fit to raters in certain conditions, it didn’t match well (ICC = 0.68) when using data from the subset of conditions (firm surface only). Contrary to the result of Brown et al., our system’s mBESS scores are able to accurately fit the rater’s (*r* = 0.92, *p* < 0.05) in the subset of balance conditions (firm surface only) as well. The support for the mBESS test of our method implies a time-saving test of postural stability when required. Moreover, the methodology by Brown et al didn’t take into consideration the error of eye opening, which results in further discrepancy relative to the BESS standard.

Dave’s study [24] using Kinect sensor V1 can only recognize three balance errors out of a total of six BESS test errors. In our study, we expanded on Dave’s work by using a second Kinect sensor which tracked the six total BESS errors, resulting in system-derived scores with accurate fit to raters (*r* = 0.93, *p* < 0.05) compared to Dave’s (*r* = 0.38) [24]. Furthermore, in Dave’s study, the subject’s balance loss may introduce unwanted error detection or may not detect errors during the BESS test, due to the joints’ location outside the FOV of the Kinect sensor. In this regard, our work suggested a method to determine the advisable sensor configuration size and hence successfully removed the problem. Moreover, Dave only used a single Kinect sensor V1 and found the camera was limited to detect eye opening during the completion of each balance trial [24]. However, the use of the Kinect V2 improved upon the its predecessors’ capability and was able to detect eye-opening, potentially as a result of an improvement of the Kinect V2 camera’s resolution from 640 × 480 to 1920 × 1080 pixels of color image, and from 320 × 240 to 512 × 424 pixels of depth image. These results suggest that the proposed methodology is an improvement over previous attempts at automating error counting while participants complete the BESS.

In addition, Napoli et al. developed an automated assessment of postural stability (AAPS) algorithm based on a single Kinect Sensor V2 to evaluate the BESS errors [23], in which low AAPS performance levels were detected in single-leg and tandem stances on foam. In a separate paper on the same work, they reported the issues detecting the back leg that is hidden behind the other leg during the tandem stance [35]. Their works were only validated by comparing the level of agreement of system’s BESS scores with that of rates and a professional camera. In our study, however, the aforementioned limitations have been addressed and verified by concurrent validity and test–retest reliability metrics.

Our study was limited to healthy normal subjects with a mean BESS score of 11.83 ± 7.62 errors. The thresholds used in the error detection algorithm, which influences the sensitivity of the BESS scoring, were determined through experiment and selection of optimal values. Therefore, the threshold values should be further considered when applied to the concussed participants. Our future research will also validate the duplex Kinect system in a clinical setting to assess errors in a large amount of concussed samples.

## 5. Conclusions

In the current study, we presented a novel Balance Error Scoring System by using duplex Kinect sensors and a custom-made algorithm. Our approach overcomes the self-occlusion problem of a previous solution using the Kinect sensor, realizing the recognition of balance error and the automatic administration of the BESS test, with a stronger test–retest reliability and concurrent validity compared to previous works. The current methodology provides a contactless clinic-based concussion administration and scoring approach that accurately detects all balance errors as per BESS instructions. Our method could be used as an affordable, portable and reliable tool for the concussion assessment in the field.

## Figures and Tables

**Table 1:** The height of the subject and the required minimal camera to subject distance.

Height *h* (m)	MCSD *d* (m)
2.1	2.65
2.2	2.82
2.3	2.99
2.4	3.12
2.5	3.32
2.6	3.49
2.7	3.67
2.8	3.83
2.9	3.99

**Table 2:** The weight coefficient for error recognition fusion.

i	j	ω(i,j)
Not tracked	Not tracked	0
Not tracked	Inferred	0
Not tracked	Well tracked	0
Inferred	Inferred	0.25
Inferred	Well tracked	0.5
Well tracked	Inferred	0.5
Well tracked	Well tracked	1

**Table 3:** The statistical results of the system and rater scores with Pearson coefficient value of each condition (*p* < 0.05).

Balance Condition	System Score	Rater Score	Pearson Coefficient *r*
Double feet firm	0.10 ± 0.40	0.10 ± 0.40	0.78
Single foot firm	3.37 ± 2.38	3.10 ± 2.34	0.89
Tandem firm	1.20 ± 2.34	1.23 ± 2.50	0.93
Double feet foam	0.33 ± 0.61	0.10 ± 0.30	0.55
Single foot foam	4.43 ± 3.13	4.30 ± 3.19	0.82
Tandem foam	2.37 ± 2.85	2.50 ± 3.08	0.87
BESS total	11.83 ± 7.62	11.33 ± 7.89	0.93
mBESS total (only firm)	4.67 ± 3.13	4.43 ± 3.40	0.92

**Table 4:** The statistical results of system score on the first and eighth day as well as the ICC value of each condition.

Balance Condition	Day 1	Day 8	ICC	*p*
Double feet firm	0.13 ± 0.30	0.10 ± 0.30	0.83	<0.001
Single foot firm	3.37 ± 2.36	3.67 ± 2.95	0.78	<0.001
Tandem firm	1.23 ± 2.36	0.93 ± 2.18	0.57	0.012
Double feet foam	0.33 ± 0.61	0.23 ± 0.50	0.58	0.010
Single foot foam	4.57 ± 3.21	4.40 ± 3.22	0.68	0.001
Tandem foam	2.40 ± 2.89	2.10 ± 2.93	0.87	<0.001
BESS total	12.07 ± 7.75	11.47 ± 6.93	0.81	<0.001
mBESS total (only firm)	4.73 ± 3.17	4.70 ± 3.76	0.84	<0.001
